# AGHE Program of Merit: Practical and Technical Application Information and Assistance

**DOI:** 10.1093/geroni/igaa057.1735

**Published:** 2020-12-16

**Authors:** Marilyn Gugliucci, Donna Weinreich

## Abstract

At a time in education when it is important for programs at colleges and universities to be productive in teaching, student recruitment, and income generation, the AGHE Program of Merit review process can aid in advancing gerontology and health professions programs. The AGHE Program of Merit has expanded its review. Now Colleges of Osteopathic Medicine and all Health Professions program may apply for Program of Merit status.. The POM designation provides gerontology programs and those health professions program that include geriatrics/gerontology content in the curriculum with an AGHE “stamp of approval” which can be used to verify program quality to administrators, to lobby for additional resources, to maintain a quality program, to market the program, and to recruit prospective students into the program. This session will begin with the “Why,” “What” and “How” of applying for Program of Merit and then provide time for small group consultation regarding the Program of Merit application. Preparation of the self-study document will be detailed by the experienced faculty members followed by the review process and timelines associated with application process. Free consultation available!

